# A systematic literature review of simulation models for non-technical skill training in healthcare logistics

**DOI:** 10.1186/s41077-018-0072-7

**Published:** 2018-07-27

**Authors:** Chen Zhang, Thomas Grandits, Karin Pukk Härenstam, Jannicke Baalsrud Hauge, Sebastiaan Meijer

**Affiliations:** 1 School of Engineering Sciences in Chemistry, Biotechnology and Health, Royal Institute of Technology, 2010, Röntgenvägen 1, 14152 Huddinge, Sweden; 2 School of Engineering Sciences in Chemistry, Biotechnology and Health, Royal Institute of Technology, Hälsovägen 11, 14152 Huddinge, Sweden; 3 Pediatric Emergency Department, Karolinska University Hospital, Tomtebodavägen 18a, 17177 Stockholm, Sweden; 4 Department of Learning, Informatics, Management and Ethics, Karolinska Institute, Tomtebodavägen 18a, 17177 Stockholm, Sweden; 5 School of Industrial Engineering and Management, Royal Institute of Technology, Mariekällgatan 3, 15144 Södertälje, Sweden

**Keywords:** Quality, Safety, Logistical simulations, Non-technical skills

## Abstract

**Background:**

Resource allocation in patient care relies heavily on individual judgements of healthcare professionals. Such professionals perform coordinating functions by managing the timing and execution of a multitude of care processes for multiple patients. Based on advances in simulation, new technologies that could be used for establishing realistic representations have been developed. These simulations can be used to facilitate understanding of various situations, coordination training and education in logistics, decision-making processes, and design aspects of the healthcare system. However, no study in the literature has synthesized the types of simulations models available for non-technical skills training and coordination of care.

**Methods:**

A systematic literature review, following the PRISMA guidelines, was performed to identify simulation models that could be used for training individuals in operative logistical coordination that occurs on a daily basis. This article reviewed papers of simulation in healthcare logistics presented in the Web of Science Core Collections, ACM digital library, and JSTOR databases. We conducted a screening process to gather relevant papers as the knowledge foundation of our literature study. The screening process involved a query-based identification of papers and an assessment of relevance and quality.

**Results:**

Two hundred ninety-four papers met the inclusion criteria. The review showed that different types of simulation models can be used for constructing scenarios for addressing different types of problems, primarily for training and education sessions. The papers identified were classified according to their utilized paradigm and focus areas. (1) Discrete-event simulation in single-category and single-unit scenarios formed the most dominant approach to developing healthcare simulations and dominated all other categories by a large margin. (2) As we approached a systems perspective (cross-departmental and cross-institutional), discrete-event simulation became less popular and is complemented by system dynamics or hybrid modeling. (3) Agent-based simulations and participatory simulations have increased in absolute terms, but the share of these modeling techniques among all simulations in this field remains low.

**Conclusions:**

An extensive study analyzing the literature on simulation in healthcare logistics indicates a growth in the number of examples demonstrating how simulation can be used in healthcare settings. Results show that the majority of studies create situations in which non-technical skills of managers, coordinators, and decision makers can be trained. However, more system-level and complex system-based approaches are limited and use methods other than discrete-event simulation.

## Background

Quality and safety in healthcare depend on the successful interaction between multiple teams, individuals, and support processes aimed at making the right resources, such as medications, medical equipment, information, and people, available at the right time [1, 2]. Furthermore, in many healthcare settings, resource utilization must be prioritized such that the person most in need of a resource from a medical perspective will receive it. The cost of failure is high, both in terms of personal tragedies as well as the socio-economic burden of increased costs due to prolonged treatments or hospital stay [3].

Many of the everyday decisions regarding how resources will be used for patient care are made by individuals and networks of people performing coordinating functions, in the sense that they manage the timing and execution of many care processes of multiple patients. Their decisions often depend on judgements combining perspectives on the relevant medical conditions, the resources at hand, and the urgency of the situation; their decisions also depend on receiving information to help make sense of the situation as well as managing high stakes and competing goals [4].

Little is known about how these prioritizing and coordination skills are learned, how people performing them build their mental system models, what information and strategies they use, and which work practices are most successful. Most of the individuals performing coordination tasks are trained on the job in an unsystematic manner, and the knowledge remains, for the most part, tacit.

Simulation in healthcare is well known as a method for training individuals and teams in escalating situations surrounding individual patients [5]. To create meaningful simulations for training the non-technical skills used in coordination [6], there is a need to develop simulations of logistical challenges in a systematic manner as well as to describe and develop learning outcomes for the non-technical skills used in coordination. To support this development, it is important to know what types of logistical problems can be addressed by what types of simulations.

Logistics is one of many growing fields in healthcare management. This trend is driven by various societal impacts; population growth and an aging society have already put pressure on the operation of healthcare systems [7, 8]. While healthcare logistics has been defined in various ways by researchers, in this paper, we define it as “operational handlings for the delivery of care, including its supportive services, from origination to recipient.” Focusing on the recipient of services, healthcare logistics could be patient-centric or material-centric. Patient-centric logistics relate to patient flows through the healthcare system. In this context, quality, safety, and efficiency of services for patients are keywords. Material-centric logistics address the positioning, storage, and circulation of goods and materials, such as blood and pharmaceutical products, within the hospital or the healthcare system.

Computer-based simulation plays an important role in the operational support of healthcare logistics. Generally, simulation can be useful in the design of complex social-technical systems [9]. As an innovative technology for adding analytical capacity, simulation can be used as an intermediate test in the (re)design of organizational rules and structures, workflow process management, performance, and avoidance of human errors [10, 11]. More specifically, according to Jun et al. [12], simulation could provide benefits such as more effective redesign or innovation, deeper insights into barriers and incentives to adoption, and provision of an environment to “bench-test” final products prior to formal release. A change or improvement in real systems, however, might be expensive or dangerous, and balancing resource allocations is a central non-technical skill for healthcare professionals. Simulation adds value by providing a solution for training individuals to solve customized problems in a virtual, persuasive environment.

The application of discrete-event simulation in healthcare began to grow considerably at the end of the 1990s [12]; however, it remains unknown what type of simulations could be used to train, develop, and test non-technical aspects of coordination. Many types of simulation paradigms exist today. Discrete-event simulation, system dynamics, and agent-based simulation are the most utilized tools for modeling and analyzing systems according to the user’s interests and the specific task addressed. Discrete-event simulation is a tool for assessing the efficiency of delivery structures, forecasting changes in patient flow and examining resource efficiency in staffing [12]. System dynamics focus on the effect of structure on behavior [13]. Instead of addressing individual transactions, system dynamics is commonly used for higher level problems, such as strategic decision making, management controls, or policy changes [14]. Agent-based simulation is based on a “bottom-up” construction for the provision of emergent phenomena based on individual interactions of resource units [15].

Literature reviews have been conducted with explicit focus on the application of simulation in patient flow or material flow. However, previous literature reviews have been limited in at least one of the following aspects: (1) Reviews usually address simulation of healthcare logistics in a very narrow manner, analyzing a single key aspect such as low stakeholder engagement [16], a single simulation technique [17], or a single department; (2) most reviews have examined papers published before 2012.

This study is a continuation of the work by Dieckmann et al. [6], with a focus on the identification of available simulation models to provide meaningful training of non-technical skills in healthcare logistics. This is the perspective through which the literature was reviewed and understood. Given the large number of training simulations published, it is of interest to explore the diversity in this genre. The objective of this study is to provide a systematic literature review to answer the following research question:

1. What types of simulation models are currently available for training non-technical skills in handling logistical issues?

## Methods

### Search strategy

To answer the research question, the Web of Science Core Collection, the ACM Digital Library, and JSTOR were searched to retrieve articles focusing on simulation in healthcare logistics between 1998 and 2017. We utilized papers from these three databases because all of them rigorously select core journals and the keynote proceedings of conferences. The search terms were divided into the following two categories: patient-centric queries and material-centric queries. The papers were screened following the Preferred Reporting Items for Systematic Reviews and Meta-Analysis (PRISMA) guidelines.

The keywords were formulated by the individual reviewers to identify papers on relevant simulation techniques and investigated systems, as summarized in Table 1. Keywords such as “healthcare,” “patient flow,” “pharma*,” “blood,” and “drug” specify the issues addressed. Keywords such as “simulation,” “system dynamics,” “simulator,” and “game” specify the research methods implemented.

**Table 1:** Queries used for the different databases

Database	Patient-centric queries	Material-centric queries
Web of Science Core Collection	TI = ((“healthcare” OR “health SAME care”) AND (“system SAME dynamics” OR “patient SAME flow” OR “gam*”)	TI = (“healthcare” OR “health SAME care OR care”) AND TS = (“pharma*” OR “blood” OR “drug”) AND TI = (“simulation” OR “system SAME dynamic*” OR “simulator*” OR “gam*”)
ACM	recordAbstract:(+(“health care” “healthcare”) + (“system dynamics” “patient flow” “gam*”))	recordAbstract:(+(“hospital” “drug” “pharma*” “blood”) + (“simulation” “simulator*” “gam*”))
JSTOR	ti:(“healthcare” OR “health care”) AND (“system dynamics” OR “patient flow” OR “game” OR “simulation”)	ti:((“drug” OR “hospital” OR “blood” OR “pharmaceutical”) AND (“system dynamics” OR “patient flow” OR “game” OR “simulation” OR “simulator”))

SAME, OR, and AND are logic operators of keywords

### Paper inclusion criteria

**Fig. 1:**
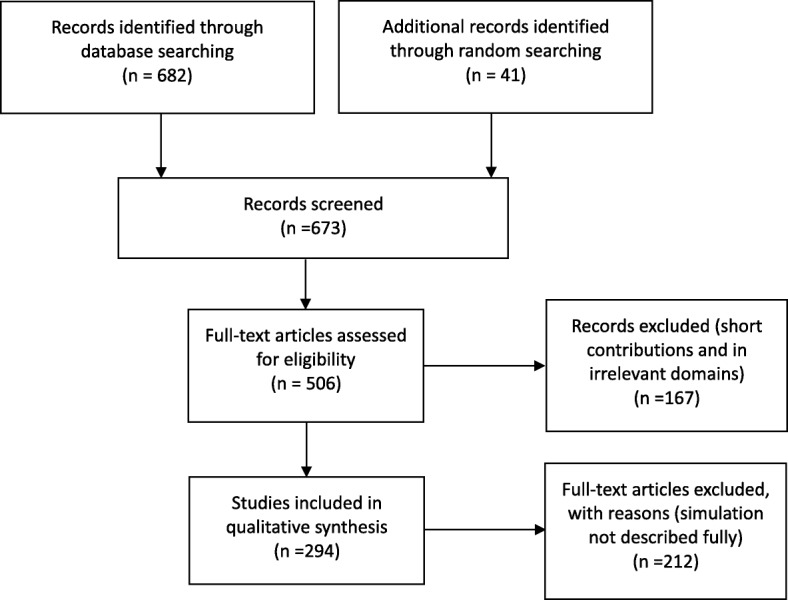
PRISMA flow diagram of assessment procedure and results: number of records included and excluded and reasons

The criteria for inclusion in this review were that studies addressed the research question and strive to improve the performance of healthcare logistics. As the focus was logistical issues in healthcare management, publications regarding epidemiology, nutrition process improvement, and statistical analysis of health programs were not included. Abstracts, book reviews of limited length, and papers not granting access to full texts were also discarded. In addition to these general requirements, criteria for classification were implemented:

1. Application-oriented paper. The paper employs at least one simulation technique and presents a detailed scenario, or experiment, of a real-world healthcare system.
2. Subjective and methodological paper. The paper focuses on subjective and methodological perspectives on simulation techniques but might not report a use case (Fig. 1).

The scope of the research and focus area were decided after the screening process. Simulation paradigms were classified based on statements from the authors; if no simulation technique was stated, the conceptualization framework was checked to determine its relevant category.

## Results

Following the retrieval of papers, discarding of duplicates, and review by the authors, the total number of essential publications was 294. The search identified 248 patient-centric and 46 material-centric papers. The patient-centric spectrum included 214 problem-solving papers, among which 114 utilized discrete-event simulation. For material-centric papers, discrete-event simulation was the dominant simulation paradigm as well. The number of publications for the past 5 years remained high, reinforcing our supposition that there is much knowledge to be gained from recent publications. The repository is available in the declaration.

For qualitative analysis, representative papers, listed in Table 2, were identified. The papers featured statements of the relevant research questions or a description of the investigated system. We considered the number of publications utilizing different simulation techniques, scopes of research, and tools.

**Table 2:** Catalog of papers

Focused issue	Reference	Paradigm	Scale	Software	Representative main finding
Care pathway and appointment	[19–21, 25, 26, 28, 29, 43, 47, 50–87, 88–127]	DES (63); ABS (3); SD (3); mixed (4), Misc. (16)	Single department (65), Cross-departments (17), cross-institutional (7)	Arena (30); Simul8(5); FlexSim (5); AnyLogic (3); NetLogo (2); Witness (2); ProModel (1); C++(1); ProcessModel (1); Microsoft Excel (1); iThink (1); AutoMod (1); SLX (2); EDSim (1); Matlab (1); DGHPSim (1); ARIS (1); MedModel (3); OMNeT++ (1); Misc. (27)	These studies focus on modeling patient pathways from admission to discharge as acting the basis of direct intervention on patient flows.
Staffing decision making	[24, 35, 37, 42, 46, 49, 55, 128–202, 248]	DES (50); SD (3); ABS (3); gaming (1); mixed (26), Misc.(5)	Single department (56), cross-departments (22), cross-institutional (10)	Arena (22); FlexSim (7); AnyLogic (5); Simul8(3); MedModel (3); FDI (3); Matlab (2); ProModel (2); Tecnomatix Plant Simulation (2); ARCINFO (1); AutoMod (1); C++ (1); Petri Nets (1); Extend (1); Microsoft Excel (1); Netlogo (1); OMNeT++(1); Venism (1); SLX (1); SIMPROCESS (1); STELLA (1); ABFS (1); Java IDE (1); Misc. (27);	These studies use simulation for decision support of care capacities.
Work procedures	[203–214]	DES (8); Misc. (4)	Single department (7), cross-departments (5)	Arena (3); Simul8(2); ProModel (1); MedModel (1); OMNeT++ (1); ExtendSim (1); Misc. (3)	Simulation is used to identify impact factors in service procedures.
Specialized Transport	[215–222]	DES (2); ABS (1); Misc. (5)	Cross-institutional (8)	Arena (2); ArcGIS (1); Google Cloud (1); Microsoft Excel (1); Misc. (3)	These studies address handling of patients in the regional healthcare network.
Facility design	[223–230]	DES (4); ABS (2); mixed (1); Misc. (1)	Single department (2), cross-departments (1)	Unity (1); ProModel (1); NetLogo (1); Extend (1); Misc. (4)	These studies use simulations to analyze hospital infrastructure and its impact on the operation.
Healthcare systems	[231–235]	DES (1); ABS (2); Misc. (2)	Cross-institutional (5)	Arena (2); Python (1); AnyLogic (1); NetLogo (1)	These studies use simulations to support the modeling and analysis of improvements in the system perspective.
Supply chain	[236–243]	DES (5), gaming (1); Misc. (2)	Cross-departments (5), cross-institutional (3)	ExtendSim (1); GAMS (1); Matlab (1); Bonita Open Solution (1), Board game (1); Misc. (3)	The simulation model is generally used for recreating different actors in the supply chain network.
Inventory management	[244–258]	DES (8); mixed (2); Misc. (5)	Single department (3), cross-departments (1), cross-institutional (11)	Simul8 (2); Arena (2); C++(1); CSIM18 (1); Java (1); JSL (1); SCA (1); Misc. (6)	These studies explore different inventory or replacement polities for material handling.
Network distribution and dispatching	[32, 259–264]	DES (4); gaming (2); ABS (1); Misc. (3)	Cross-departments (2), cross-institutional (8)	Microsoft Excel (2); Arena (1); MedModel (1); ProModel (1); JADE (1); VBA (1); Misc. (3)	These studies use simulations for operational transport.
Network configuration	[265–267]	DES (1); Misc. (2)	Cross-institutional (3)	Arena (1); Misc. (2)	These studies focus on the design of the network.
Procurement logistics	[27, 268, 269]	SD (1); Misc. (2)	Cross-department (1), cross-institutional (2)	Qnet2000 (1); iThink (1); Misc. (1)	Simulation is used for understanding the interactive rule between service vendor and recipient.
Misc.	[31, 36, 38, 270–281]	DES (4); SD (3); ABS (1); gaming (1); mixed (3); Misc. (4)	Single department (5), cross-departments (4), cross-institutional (7)	Arena (4); AnyLogic (2); iThink (2); Simul8 (1); NetLogo (1); Microsoft Excel (1); Powersim (1); Misc. (4)	
Methodology	[12–15, 39, 282–286, 287–305]				Reviews, surveys, and methodological reflections and comparisons of logistics simulations in other sectors.

Following the screening process, we identified the question levels and the categories of addressed issues. The following question levels were derived: single department/unit, cross-department/unit, and cross-institutional. The category single department/unit included studies that model operation within a single department in an organization. The category cross-department/unit included studies that simulate multiple departments/units within the same organization. The category cross-institutional included the simulation modeling of interactions and flows between healthcare service providers in a large-scale network with widespread distribution regions. We identified the following categories of addressed issues: care pathway and appointment, staffing decision making, work procedures, specialized transport, facility design, healthcare systems, supply chain, inventory management, network distribution and dispatching, network configuration, procurement logistics, methodological contributions, and miscellaneous. The facility design was considered because architectural planning is a strategic decision that has a durable and profound effect on healthcare operation. The miscellaneous category included all research publications that we were not able to clearly classify into at least one of the abovementioned categories.

### Logistical simulations—review

#### Discrete-event simulation

Discrete-event simulation has been applied to model and analyze all aspects of logistics management in healthcare. In particular, patient flow management and planning of staffing requirement are effective applications of this simulation technology. Our profiling of the literature is mostly in line with the findings of previous literature reviews; that is, discrete-event simulation is a useful tool with respect to improving patient flow, managing bed capacity, and scheduling and utilizing of resources [16, 17]. DeRienzo et al. addressed the effect of nursing capacity by comparing different nursing sizes and demonstrated the applicability of supporting healthcare managers in handling operative tasks [18]. Devapriya et al. also developed a decision-supporting tool based on discrete-event simulation for the strategic planning of hospital bed capacity [19]. Bhattacharjee et al. analyzed appointment scheduling policies for patients to be treated by a medical scanning machine [20]. Vasilakis et al. developed a discrete-event simulation to study how long it took for patients to obtain their appointments from their referral [21]. Jørgensen et al. investigated internal blood logistics in hospitals and evaluated the effects of various management controls on the waiting times for accessing blood samples [22]. This simulation paradigm is most suitable for the realistic representation of processes in health services for analyzing “what-if” scenarios and assessing the performance of a logistical system.

#### System dynamics

System dynamics is used for organizational simulations. The paradigm is a mechanism-driven one for making decisions strategically for health services and resources from a global perspective. For instance, Rashwan et al. developed a system dynamics simulation to study bed blocking in Irish hospitals [23]. The focus was twofold: testing the policies for solving delayed discharges and envisaging the counterproductive and unintended consequences of these new policies [24]. Brailsford et al. simulated patient flow perspectives to identify system-wide bottlenecks [25]. Through the simulation, Lane et al. showed that the daily variation of used hospital bed capacity could not be balanced in the long run by simply increasing capacity; instead, optimal design of flows should be the core of the operation technology [26]. One paper investigated logistical outsourcing [27] and deployed system dynamics simulation with a sensitivity analysis for the evaluation and analysis of sustainability and economic performance. Content holders can use system dynamics simulation to envisage the complexity and identify opportunities and risks of the policies and management controls proposed.

#### Agent-based simulation

Agent-based simulation could be considered a means of soft computing in healthcare logistics. Agent-based simulation provides a gateway for understanding the behavior of distributed and connected service providers. The associated modeling and analysis are able to handle engineering system problems in complex networks. As an example, this paradigm was introduced to solve the coordination and collaboration difficulty of caregivers in a mental healthcare system [28]. The positive effect of coordination technology was confirmed by such modeling considerations. The local decision rules of caregivers were relevant for operative decision making with respect to successful provision of home help, a conclusion drawn from Marcon et al.’s work [29]. Bidding decisions made by distributers and suppliers in the pharmaceutical industry were studied in Jetly et al.’s work [30]. The performance of a multi-site network was simulated with pre-selected indicators, including the number of released products, degree of consolidation, and the return on assets. Multi-agent systems are not only effective for modeling flows between providers; they could also be applied in hospital environments. In Marin et al.’s work, patients, nurses, doctors, and the department as the manager are specified as agents with simple behavior rules [31]. With the support of multi-agent languages, the properties and relationships of actors could be simulated and validated for a specific social-technical environment.

#### Game and participatory simulation

Games and participatory simulation are life-like media that facilitate experimental learning. The use of such media enables the development of non-technical teamwork skills. For instance, Mustafee and Katsaliaki developed a pedagogical business game that simulated the blood supply chain [32, 33]. The players were encouraged to propose different solutions, taking costs, time-efficiency, and stock levels of products into account. Focusing on quality of service in healthcare, a web-based organizational simulation was built and deployed for training referral and diagnostic skills [34]. The results showed that the usefulness of information on symptoms, diseases, and severity levels is associated with the perception of information sources. A board game provided valuable insight into the adoption of future technology in hospital logistical work [15]. Regarding the practical settings of two hospitals, the adoption of wearable technologies was reflected through role play. Such role playing could also be used to analyze the working environment in wards [35].

#### Hybrid modeling

Hybrid modeling is the efficient combination of various simulation modeling techniques described. Most hybrid modeling involves coupling discrete-event simulation and system dynamics. Two case studies, concerning infection control and regional social care system engineering, were simulated using hybrid models [36]. Zulkepli modeled an integrated ICU by combining system dynamics and discrete-event simulation [37]. The greatest advantage of hybrid modeling is the ability to integrate different simulation approaches and empirical data from different sources [38].

### Analysis of trends

**Fig. 2:**
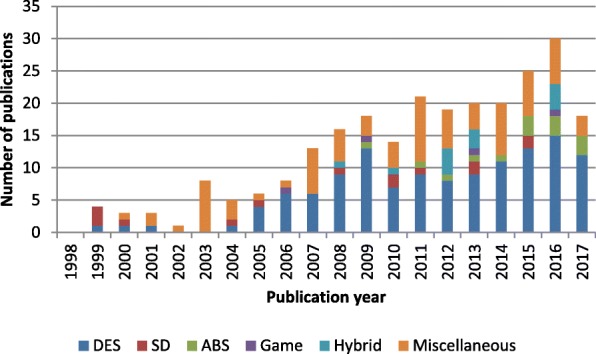
Simulation paradigms for patient-centric logistics

Discrete-event simulation has been the most prominent paradigm for modeling patient-centric logistics over the past decade. Between 2008 and 2017, as presented in Fig. 2, more than half of the included papers used discrete-event simulation. However, the distribution of simulation techniques among different periods showed growing methodological diversity in recent years. The presence of system dynamics is observed in all periods, although the number of publications remained small. Agent-based simulation, games, and hybrid modeling were utilized only in the last decade. The specific simulation paradigm used was not stated in some studies, especially between 1998 and 2007, during which the majority of the methods used were classified as miscellaneous. Game-based methods were used in a few studies. Thus, interactive simulations are still quite new and rarely used.

**Fig. 3:**
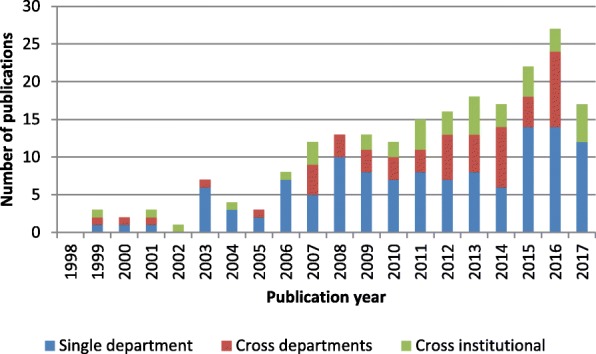
Research scope for patient-centric logistics

The single category was the predominant level addressed in all periods, as shown in Fig. 3. Between 2008 and 2012, the majority of studies addressed logistical issues at the single-unit level. The systems perspective was introduced between 2013 and 2017. Work addressing logistics issues at the cross-departmental and cross-institutional levels formed half of all research efforts. However, cross-institutional issues remained largely underexplored in the literature compared with other problems studied.

**Fig. 4:**
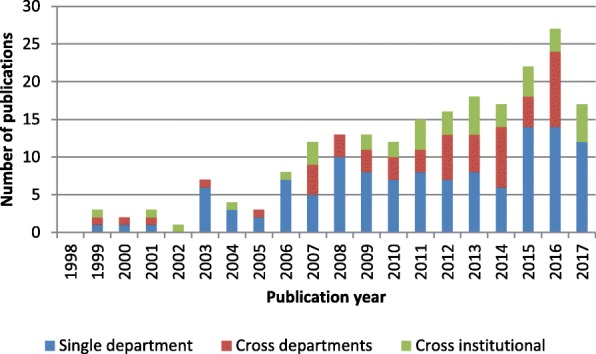
Simulation paradigms for material-centric logistics

Discrete-event simulation was the most prominent simulation paradigm in material-centric approaches as well, as shown in Fig. 4. The research theme started to develop in 2006, after which the number of publications and the diversity of the utilized paradigms increased. Despite the growth, six of 16 papers utilized discrete-event simulation, and only three papers utilized system dynamics, agent-based simulation, and/or hybrid modeling.

Compared with patient-centric logistics, material-centric logistics was covered by a limited number of articles. Shah et al. had already stressed the underexplored potential of this area in 2004 [39]. We identified few publications on this subject during this period. The period 2013–2017 showed the largest output, but the volume was still not able to catch up with that of papers related to patient-centric logistics.

**Fig. 5:**
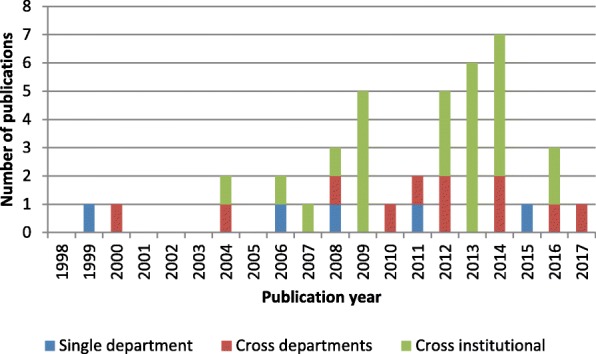
Research scope for material-centric logistics

As shown in Fig. 5, a growing number of papers have analyzed material handling between multiple units. However, simulation design for cross- and single-department logistics was lacking over the last 5 years, despite studies reporting on the need for improving hospital internal supply chains to reduce costs [40].

## Discussions

The literature review demonstrated that different simulation techniques could be utilized for different educational purposes, as summarized in Table 3. Discrete-event simulation is suitable for operational problems, whereas strategic issues are better explored by system dynamics. Agent-based simulation stands out as a versatile tool because that agent method is object-oriented and flexible for describing the anatomy of complex systems formed by multiple actors. Healthcare logistics is a complex socio-technical system characterized by interconnected components and non-rational operation management. Agent-based simulation can explicitly model the interaction between system components, facilitating the understanding of overall performance under uncertainty and dynamics.

**Table 3:** Guidelines for selecting a suitable logistical simulation model for training purposes

	Discrete-event simulation	System dynamics	Agent-based simulation	Game and participatory simulation
Level of training	Operational	Strategic	All	Tactical, operational
Training purpose	Process management and innovation	Planning	Reasoning, negotiation, distributed management	Experience, awareness, perception
Lower boundary of technical preparation	Qualitative workflow	Casual loop	Objected-oriented programming	Low-tech material
Higher boundary of technical preparation		Differential equations	Agent system	High-tech graphic and interaction
Applicable area	All	Staffing decision making, procurement logistics	Staffing decision making, transport, hospital design, network distribution and dispatching	Staffing decision making, supply chain management, network distribution and dispatching
Tools	Arena, Simio, Simu8, AnyLogic	Venism, AnyLogic	NetLogo, AnyLogic	Boards, unity

Games and participatory simulation are particularly useful for training at the tactical level because games help identify productive or counterproductive human actions. The strength of the agent-based method is the modeling and analysis of human behavior [33, 41]. Healthcare logistics are largely characterized by non-rational operative decision making by medical personnel regarding needs of their patients. By modeling decisions at the agent level, it is possible to obtain insight into the reasoning process of decisions being made [42]. By involving these operational experts in participatory simulations, we can assess their perception of processes and healthcare system operations [43]. This effort delivers insight at another level of abstraction than technical, often discrete-event-based, simulations can provide.

Regarding training purposes, agent-based simulation and games are suitable for training negotiation and coordination in logistics, whereas discrete-event simulation and system dynamics can be utilized for reducing the uncertainty of decision-making processes by adding details to the model.

Participatory simulation is valuable for validating various simulators that model complex systems. The advantage of participatory simulation corresponds to the delivery structure of the investigated system, as healthcare logistics is carried out by collaborative efforts in which different professionals, knowledge, and skills work together.

Discrete-event simulation has the lowest requirement of technological preparation and is found to support all areas [26, 44–46]. System dynamics and agent-based simulation might require formal methods and mathematics pertaining to system design, such as differential equations [47], decision theories [48], and game-based approaches [33].

## Conclusions

Complex socio-technical systems, such as air traffic controls [49], routinely apply flow and logistical simulations. The studies examined in this review indicate a growing practice of implementing simulation in healthcare settings to create situations in which the non-technical skills of managers, coordinators, and decision makers can be trained and developed. Building on existing concepts from other industries [50], future applications might be hands-on training of teams using gaming and participatory simulation alongside empirical data to create situations for training tricky decision making, for strategic planning, and for exploring the effect of decisions on other parts of the system [51].

Our review yielded many examples of applications in healthcare, indicating that the issues of training of strategic or operational coordination and decision making in healthcare can all be addressed by simulation. The orthogonal simulation techniques are discrete-event simulation, system dynamics, agent-based simulation, game, and participatory simulation. For patient-centric logistics, discrete-event simulation in single-department/unit scenarios is the most dominant form of simulation, the maturity of which takes the lead over other categories by a large margin. As a systems perspective was applied, discrete-event simulation became less popular and was compensated for by system dynamics or hybrid modeling. The literature review showed that tools for logistical simulations vary in this field, with tools such as AnyLogic, Arena, NetLogo, and board games implemented most frequently. This is an extensive study analyzing the growth in the use of simulation in healthcare settings.

### Lack of standardization

The number of miscellaneous simulations was significant, although discrete-event simulation, system dynamics, and agent-based simulation were well-established and well-standardized simulation techniques in many software packages. Most of the miscellaneous simulations were custom-made solutions. A focal point of these papers was implementing the modular design of protocols, revealing a lack of standards. Compared with processes in many other industries, healthcare processes are less standardized, and thus, composition of services varies. We believe that much effort could be saved by employing standardization in both healthcare processes and simulation formulism.

### Lack of identification for material-centric logistics

In the domain of material-centric logistics, the focus is on inventory management and network distribution. A general lack of articles indicated limited research effort. One reason is that material-centric logistics is not an independent research stream yet—in many cases, the analysis of material-centric logistics is attached to a larger research project pertaining to physical distribution and logistical management.

### Lack of complex system modeling and simulation

System dynamics, agent-based simulation, and hybrid modeling were underdeveloped for handling the complexity of social-technical systems. Digital transformation would change many aspects of the human-technology interaction in the provision of health services. A knowledge gap exists between the promise of future delivery of care that abolishes institutional boundaries and the current methods for testing and demonstrating functionalities. To bridge this gap, we require a better understanding of interconnected relationships between care providers and extensions to model individual-level requirements.

### Limitations

The review has limitations. The search terms were formulated by the authors. As a result, the data search might not have been comprehensive. To eliminate the risk of omitting important contributions, the search terms combined keywords related to content and scientific methods, respectively. Second, although both journal and conference contributions were considered, the exclusion of abstracts and posters might lead to publication bias according to the Assessment of Multiple Systematic Reviews (AMSTAR) checklist for assessing the quality of systematic reviews. Because the aim of the review was to identify logistical simulations for training and education purposes, the exclusion is understandable for the short contributions that are not able to document the simulation models in a detailed manner. Therefore, publication bias is not prevalent in our literature review. The review only analyzed papers published since 1998. This approach was taken because the growth in the use of healthcare simulations started as Jun et al. surveyed the practical application of discrete-event simulation in healthcare [12], which was noted by Persson and Persson [52]. Therefore, a synthesis of the literature after 1998 should not distort the analysis.

### General conclusion

The overview demonstrates that the simulation models available are mainly event-based, which is understandable. The strict regulations and rules associated with the medical field make process simulation particularly suited to handling issues in this area. These perceptions, together with the lack of literature on using agent-based simulation and participatory simulation, suggest a research direction involving the development of ontologies, architectures, and terminologies for their better acceptance in training and education of non-technical skills, with more problem-solving studies performed to demonstrate the corresponding benefits.

It is worth noting that the growth of digitalized healthcare occurs in parallel with the demographic change into an aging society. Currently, digital transformation, provision of homecare, and de-institutionalization are transferring practical applications into the decentralized paradigm. This effort requires coordination between caregivers and stakeholders. Agent-based simulation and participatory simulation can support comprehensive engineering to achieve quality and safety improvements. Therefore, agent-based simulation and participatory simulation are promising approaches for better handling healthcare logistics given current societal trends.

## Abbreviations

ABS: Agent-based simulation
DES: Discrete-event simulation
Misc: Miscellaneous
Mixed: Hybrid simulation
SD: Systems dynamics

## References

[1] Klein G, Feltovich PJ, Bradshaw JM, Woods DD. Common ground and coordination in joint activity. In: Rouse WB, Boff KR, editors. Organizational simulation. New Jersey: Wiley; 2005. p. 139–84.

[2] Vincent C, Amalberti R. Safety strategies in hospitals. In: Safer healthcare. Cham: Springer International Publishing. p. 73–91.

[3] Nemeth CP, Nunnally M, O’Connor MF, Brandwijk M, Kowalsky J, Cook RI (2007). Regularly irregular: how groups reconcile cross-cutting agendas and demand in healthcare. Cogn Tech Work.

[4] Macrae C, Draycott T. Delivering high reliability in maternity care: in situ simulation as a source of organisational resilience. Saf Sci. 2016. 10.1016/j.ssci.2016.10.019.

[5] Crichton M, O’Connor P, Flin R. Safety at the sharp end: a guide to non-technical skills. Hampshire: Ashgate Publishing, Ltd; 2013.

[6] Dieckmann P, Zeltner LG, Helsø A-M (2016). “Hand-it-on”: an innovative simulation on the relation of non-technical skills to healthcare. Adv Simul.

[7] Ulmanen P, Szebehely M (2015). From the state to the family or to the market? Consequences of reduced residential eldercare in Sweden: from the state to the family. Int J Soc Welf.

[8] Hagihara A, Hasegawa M, Hinohara Y, Abe T, Motoi M (2013). The aging population and future demand for emergency ambulances in Japan. Intern Emerg Med.

[9] Kriz WC (2003). Creating effective learning environments and learning organizations through gaming simulation design. Simul Gaming.

[10] Kriz WC (2017). Types of gaming simulation applications. Simul Gaming.

[11] Meijer S (2015). The power of sponges: comparing high-tech and low-tech gaming for innovation. Simul Gaming.

[12] Jun JB, Jacobson SH, Swisher JR (1999). Application of discrete-event simulation in health care clinics: a survey. J Oper Res Soc.

[13] Brailsford SC (2008). System dynamics: what’s in it for healthcare simulation modelers. Proceedings of the winter simulation conference.

[14] Koelling P, Schwandt MJ (2005). Health systems: a dynamic system-benefits from system dynamics. Proceedings of the winter simulation conference.

[15] Mustafee N, Katsaliaki K, Taylor SJE (2010). Profiling literature in healthcare simulation. Simulation.

[16] Schaefer JJ, Vanderbilt AA, Cason CL, Bauman EB, Glavin RJ, Lee FW (2011). Literature review: instructional design and pedagogy science in healthcare simulation. Simul Healthc.

[17] Nestel D, Groom J, Eikeland-Husebø S, OʼDonnell JM (2011). Simulation for learning and teaching procedural skills: the state of the science. Simul Healthc.

[18] DeRienzo CM, Shaw RJ, Meanor P, Lada E, Ferranti J, Tanaka D (2016). A discrete event simulation tool to support and predict hospital and clinic staffing. Health Informatics J.

[19] Devapriya P, Strömblad CTB, Bailey MD, Frazier S, Bulger J, Kemberling ST (2015). StratBAM: a discrete-event simulation model to support strategic hospital bed capacity decisions. J Med Syst.

[20] Bhattacharjee P, Ray PK (2016). Simulation modelling and analysis of appointment system performance for multiple classes of patients in a hospital: a case study. Oper Res Health Care.

[21] Vasilakis C, Sobolev BG, Kuramoto L, Levy AR (2007). A simulation study of scheduling clinic appointments in surgical care: individual surgeon versus pooled lists. J Oper Res Soc.

[22] Jørgensen P, Jacobsen P, Poulsen JH (2013). Identifying the potential of changes to blood sample logistics using simulation. Scand J Clin Lab Invest.

[23] Rashwan W, Ragab MA, Abo-Hamad W, Arisha A (2013). Bed blockage in Irish hospitals: system dynamics methodology. Proceedings of the winter simulation conference.

[24] Rashwan W, Abo-Hamad W, Arisha A (2015). A system dynamics view of the acute bed blockage problem in the Irish healthcare system. Eur J Oper Res.

[25] Brailsford SC, Lattimer VA, Tarnaras P, Turnbull JC (2004). Emergency and on-demand health care: modelling a large complex system. J Oper Res Soc.

[26] Lane DC, Monefeldt C, Rosenhead JV (2000). Looking in the wrong place for healthcare improvements: a system dynamics study of an accident and emergency department. J Oper Res Soc.

[27] Azzi A, Persona A, Sgarbossa F, Bonin M (2013). Drug inventory management and distribution: outsourcing logistics to third-party providers. Strateg Outsourcing Int J.

[28] Kalton A, Falconer E, Docherty J, Alevras D, Brann D, Johnson K (2016). Multi-agent-based simulation of a complex ecosystem of mental health care. J Med Syst.

[29] Marcon E, Chaabane S, Sallez Y, Bonte T, Trentesaux D (2017). A multi-agent system based on reactive decision rules for solving the caregiver routing problem in home health care. Simul Model Pract Theory.

[30] Jetly G, Rossetti CL, Handfield R (2012). A multi-agent simulation of the pharmaceutical supply chain. J Simul.

[31] Escudero-Marin P, Pidd M (2011). Using ABMS to simulate emergency departments. Proceedings of the winter simulation conference.

[32] Mustafee N, Katsaliaki K (2010). The blood supply game. Proceedings of the winter simulation conference.

[33] Katsaliaki K, Mustafee N, Kumar S (2014). A game-based approach towards facilitating decision making for perishable products: an example of blood supply chain. Expert Syst Appl.

[34] Basole RC, Bodner DA, Rouse WB (2013). Healthcare management through organizational simulation. Decis Support Syst.

[35] Mattarelli E, Fadel KJ, Weisband SP (2006). Design of a role-playing game to study the trajectories of health care workers in an operating room. Proceedings of conference on human factors in computing systems.

[36] Brailsford SC, Desai SM, Viana J (2010). Towards the holy grail: combining system dynamics and discrete-event simulation in healthcare. Proceedings of the winter simulation conference.

[37] Zulkepli J, Eldabi T, Mustafee N (2012). Hybrid simulation for modelling large systems: an example of integrated care model. Proceedings of the winter simulation conference.

[38] zen MB, Zabawa J (2016). Modeling healthcare demand using a hybrid simulation approach. Proceedings of the winter simulation conference.

[39] Shah N (2004). Pharmaceutical supply chains: key issues and strategies for optimisation. Comput Chem Eng.

[40] Gaba DM (2012). Adapting space science methods for describing and planning research in simulation in healthcare: science traceability and decadal surveys. Simul Healthc.

[41] Liu Z, Rexachs D, Epelde F, Luque E (2017). An agent-based model for quantitatively analyzing and predicting the complex behavior of emergency departments. J Comput Sci.

[42] Kolb EMW, Schoening S, Peck J, Lee T (2008). Reducing emergency department overcrowding: five patient buffer concepts in comparison. Proceedings of the winter simulation conference.

[43] Kotiadis K, Tako AA, Vasilakis C (2014). A participative and facilitative conceptual modelling framework for discrete event simulation studies in healthcare. J Oper Res Soc.

[44] Demir E, Gunal MM, Southern D (2017). Demand and capacity modelling for acute services using discrete event simulation. Health Syst.

[45] Reynolds M, Vasilakis C, McLeod M, Barber N, Mounsey A, Newton S (2011). Using discrete event simulation to design a more efficient hospital pharmacy for outpatients. Health Care Manag Sci.

[46] Pehlivan C, Augusto V, Xie X (2013). Admission control in a pure loss healthcare network: MDP and DES approach. Proceedings of the winter simulation conference.

[47] Medeiros DJ, Swenson E, DeFlitch C (2008). Improving patient flow in a hospital emergency department. Proceedings of the winter simulation conference.

[48] Ferrin DM, Miller MJ, McBroom DL (2007). Maximizing hospital finanacial impact and emergency department throughput with simulation. Proceedings of the winter simulation conference.

[49] Dehlendorff C, Kulahci M, Andersen KK (2011). Designing simulation experiments with controllable and uncontrollable factors for applications in healthcare. Appl Stat.

[50] Duguay C, Chetouane F (2007). Modeling and improving emergency department systems using discrete event simulation. Simulation.

[51] Kittipittayakorn C, Ying K-C. Using the integration of discrete event and agent-based simulation to enhance outpatient service quality in an orthopedic department. J Healthc Eng. 2016;2016. 10.1155/2016/4189206 PMC5058573 27195606

[52] Persson MJ, Persson JA (2010). Analysing management policies for operating room planning using simulation. Health Care Manag Sci.

[53] Guo M, Wagner M, West C (2004). Outpatient clinic scheduling—a simulation approach. Proceedings of the winter simulation conference.

[54] Wijewickrama A, Takakuwa S (2005). Simulation analysis of appointment scheduling in an outpatient department of internal medicine. Proceedings of the winter simulation conference.

[55] Johansson B, Jain S, Montoya-Torres J, Hngan J (2010). Integrating balanced scorecard and simulation modeling to improve emergency department performance in Irish hospitals. Proceedings of the winter simulation conference.

[56] Lin Y, Zhang J (2011). An ant colony optimization approach for efficient admission scheduling of elective inpatients. Proceedings of the annual conference on genetic and evolutionary computation.

[57] Helm JE, AhmadBeygi S, Van Oyen MP (2011). Design and analysis of hospital admission control for operational effectiveness. Prod Oper Manag.

[58] Yokouchi M, Aoki S, Sang H, Zhao R, Takakuwa S (2012). Operations analysis and appointment scheduling for an outpatient chemotherapy department. Proceedings of the winter simulation conference.

[59] Khurma N, Salamati F, Pasek ZJ (2013). Simulation of patient discharge process and its improvement. Proceedings of the winter simulation conference.

[60] Kooij R, Mes MRK, Hans EW (2014). Simulation framework to analyze operating room release mechanisms. Proceedings of the winter simulation conference.

[61] Huggins A, Claudio D, Waliullah M (2014). A detailed simulation model of an infusion treatment center. Proceedings of the winter simulation conference.

[62] Prodel M, Augusto V, Xie X (2014). Hospitalization admission control of emergency patients using markovian decision processes and discrete event simulation. Proceedings of the winter simulation conference.

[63] Giesen E, Ketter W, Zuidwijk R, Müller JP, Ketter W, Kaminka G, Wagner G, Bulling N (2015). Dynamic agent-based scheduling of treatments: evidence from the Dutch youth health care sector. Multiagent system technologies.

[64] Chen Y, Kuo YH, Balasubramanian H, Wen C (2015). Using simulation to examine appointment overbooking schemes for a medical imaging center. Proceedings of the winter simulation conference.

[65] Crowe S, Vasilakis C, Gallivan S, Bull C, Fenton M (2015). Informing the management of pediatric heart transplant waiting lists: complementary use of simulation and analytical modeling. Proceedings of the winter simulation conference.

[66] Chen P-S, Robielos RAC, Palaña PKVC, Valencia PLL, GY-H C (2015). Scheduling patients’ appointments: allocation of healthcare service using simulation optimization. J Healthc Eng.

[67] Kim S-H, Chan CW, Olivares M, Escobar G (2015). ICU admission control: an empirical study of capacity allocation and its implication for patient outcomes. Manag Sci.

[68] Khanna S, Boyle J, Good N, Bell A, Lind J (2017). Analysing the emergency department patient journey: discovery of bottlenecks to emergency department patient flow. Emerg Med Australas.

[69] Kim BBJ, Delbridge TR, Kendrick DB (2017). Adjusting patients streaming initiated by a wait time threshold in emergency department for minimizing opportunity cost. Int J Health Care Qual Assur.

[70] Ozcan YA, Tànfani E, Testi A (2017). Improving the performance of surgery-based clinical pathways: a simulation-optimization approach. Health Care Manag Sci.

[71] Bozzetto M, Rota S, Vigo V, Casucci F, Lomonte C, Morale W (2017). Clinical use of computational modeling for surgical planning of arteriovenous fistula for hemodialysis. BMC Med Inform Decis Mak.

[72] Vile JL, Allkins E, Frankish J, Garland S, Mizen P, Williams JE (2017). Modelling patient flow in an emergency department to better understand demand management strategies. J Simul.

[73] Demir E, Southern D (2017). Enabling better management of patients: discrete event simulation combined with the STAR approach. J Oper Res Soc.

[74] Chepenik L, Pinker E (2017). The impact of increasing staff resources on patient flow in a psychiatric emergency service. Psychiatr Serv.

[75] Saltzman R, Roeder T, Lambton J, Param L, Frost B, Fernandes R (2017). The impact of a discharge holding area on the throughput of a pediatric unit. Serv Sci.

[76] Toth DJA, Khader K, Slayton RB, Kallen AJ, Gundlapalli AV, O’Hagan JJ (2017). The potential for interventions in a long-term acute care hospital to reduce transmission of carbapenem-resistant enterobacteriaceae in affiliated healthcare facilities. Clin Infect Dis.

[77] Steward D, Glass TF, Ferrand YB (2017). Simulation-based design of ed operations with care streams to optimize care delivery and reduce length of stay in the emergency department. J Med Syst.

[78] Tako AA, Kotiadis K (2015). PartiSim: a multi-methodology framework to support facilitated simulation modelling in healthcare. Eur J Oper Res.

[79] Monks T, Pearn K, Allen M (2015). Simulation of stroke care systems. Proceedings of the winter simulation conference.

[80] Djanatliev A, Meier F (2016). Hospital processes within an integrated system view: a hybrid simulation approach. Proceedings of the winter simulation conference.

[81] Alahäivälä T, Oinas-Kukkonen H (2016). Understanding persuasion contexts in health gamification: a systematic analysis of gamified health behavior change support systems literature. Int J Med Inform.

[82] Zhong X, Lee HK, Li J (2017). From production systems to health care delivery systems: a retrospective look on similarities, difficulties and opportunities. Int J Prod Res.

[83] Lim ME, Worster A, Goeree R, Tarride J-É (2013). Simulating an emergency department: the importance of modeling the interactions between physicians and delegates in a discrete event simulation. BMC Med Inform Decis Mak.

[84] Vanderby S, Carter MW (2010). An evaluation of the applicability of system dynamics to patient flow modelling. J Oper Res Soc.

[85] Chockalingam A, Jayakumar K, Lawley MA (2010). A stochastic control approach to avoiding emergency department overcrowding. Proceedings of the winter simulation conference.

[86] Djanatliev A, German R (2013). Prospective healthcare decision-making by combined system dynamics, discrete-event and agent-based simulation. Proceedings of the winter simulation conference.

[87] Wolstenholme E (1999). A patient flow perspective of U.K. health services: exploring the case for new “intermediate care” initiatives. Syst Dyn Rev.

[88] Jansen FJA, Etman LFP, Rooda JE, Adan IJBF (2012). Aggregate simulation modeling of an MRI department using effective process times. Proceedings of the winter simulation conference.

[89] Zambrano F, Concha P, Ramis F, Neriz L, Bull M, Veloz P (2016). Improving patient access to a public hospital complex using agent simulation. Proceedings of the winter simulation conference.

[90] Lamé G, Jouini O, Cardinal JS-L, Carvalho M, Tournigand C, Wolkenstein P (2016). Patient-hospital communication: a platform to improve outpatient chemotherapy. Proceedings of the winter simulation conference.

[91] April J, Better M, Glover F, Kelly J, Laguna M (2006). Enhancing business process management with simulation optimization. Proceedings of the winter simulation conference.

[92] Bowers J, Ghattas M, Mould G (2012). Exploring alternative routes to realising the benefits of simulation in healthcare. J Oper Res Soc.

[93] Chavis J, Cochran AL, Kocher KE, Washington VN, Zayas-Cabán G (2016). A simulation model of patient flow through the emergency department to determine the impact of a short stay unit on hospital congestion. Proceedings of the winter simulation conference.

[94] Patvivatsiri L (2006). A simulation model for bioterrorism preparedness in an emergency room. Proceedings of the winter simulation conference.

[95] Comas M, Castells X, Hoffmeister L, Román R, Cots F, Mar J (2008). Discrete-event simulation applied to analysis of waiting lists: evaluation of a prioritization system for cataract surgery. Value Health.

[96] Kuhl ME (2012). A simulation study of patient flow for day of surgery admission. Proceedings of the winter simulation conference.

[97] Miller M, Ferrin D, Shahi N (2009). Estimating patient surge impact on boarding time in several regional emergency departments. Proceedings of the winter simulation conference.

[98] Roure M, Halley Q, Augusto V (2015). Modelling and simulation of an outpatient surgery unit. Proceedings of the winter simulation conference.

[99] Doğan NÖ, Unutulmaz O (2016). Lean production in healthcare: a simulation-based value stream mapping in the physical therapy and rehabilitation department of a public hospital. Total Qual Manag Bus Excell.

[100] Batarseh OG, Goldlust EJ, Day TE (2013). SysML for conceptual modeling and simulation for analysis: a case example of a highly granular model of an emergency department. Proceedings of the winter simulations conference.

[101] Schonherr O, Rose O (2011). A general model description for discrete processes. Proceedings of the winter simulation conference.

[102] Southard PB, Chandra C, Kumar S (2012). RFID in healthcare: a six sigma DMAIC and simulation case study. Int J Health Care Qual Assur.

[103] Santibáñez P, Chow VS, French J, Puterman ML, Tyldesley S (2009). Reducing patient wait times and improving resource utilization at British Columbia Cancer Agency’s ambulatory care unit through simulation. Health Care Manag Sci.

[104] Konrad RA, Lawley MA (2009). Input modeling for hospital simulation models using electronic messages. Proceedings of the winter simulation conference.

[105] Hagtvedt R, Ferguson M, Griffin P, Jones GT, Keskinocak P (2009). Cooperative strategies to reduce ambulance diversion. Proceedings of the winter simulation conference.

[106] Maull RS, Smart PA, Harris A, AA-F K (2009). An evaluation of ‘fast track’ in A&E: a discrete event simulation approach. Serv Ind J.

[107] Roberts SD (2011). Tutorial on the simulation of healthcare systems. Proceedings of the winter simulation conference.

[108] Hosseini S, Jannat S (2015). Discrete event simulation technique for evaluating performance of oncology department: a case study. Proceedings of the winter simulation conference.

[109] Levin S, Garifullin M. Simulating wait time in healthcare: accounting for transition process variability using survival analyses. In: Proceedings of the winter simulation conference. 2015. p. 1252–60.

[110] Levin S, Dittus R, Aronsky D, Weinger M, France D (2011). Evaluating the effects of increasing surgical volume on emergency department patient access. Qual Saf Health Care.

[111] Mahapatra S, Koelling CP, Patvivatsiri L, Fraticelli B, Eitel D, Grove L (2003). Pairing emergency severity index5-level triage data with computer aided system design to improve emergency department access and throughput. Proceedings of the winter simulation conference.

[112] McClean S, Barton M, Garg L, Fullerton K (2011). A modeling framework that combines Markov models and discrete-event simulation for stroke patient care. ACM Trans Model Comput Simul.

[113] Reindl S, Mönch L, Mönch M, Scheider A (2009). Modeling and simulation of cataract surgery processes. Proceedings of the winter simulation conference.

[114] Takakuwa S, Wijewickrama A (2008). Optimizing staffing schedule in light of patient satisfaction for the whole outpatient hospital ward. Proceedings of the winter simulation conference.

[115] Takakuwa S, Katagiri D (2007). Modeling of patient flows in a large-scale outpatient hospital ward by making use of electronic medical records. Proceedings of the winter simulation conference.

[116] Takakuwa S, Shiozaki H (2004). Functional analysis for operating emergency department of a general hospital. Proceedings of the winter simulation conference.

[117] Coelli FC, Ferreira RB, Almeida RMVR, Pereira WCA (2007). Computer simulation and discrete-event models in the analysis of a mammography clinic patient flow. Comput Methods Prog Biomed.

[118] Rohleder TR, Lewkonia P, Bischak DP, Duffy P, Hendijani R (2011). Using simulation modeling to improve patient flow at an outpatient orthopedic clinic. Health Care Manag Sci.

[119] Wang T, Guinet A, Belaidi A, Besombes B (2009). Modelling and simulation of emergency services with ARIS and Arena. Case study: the emergency department of Saint Joseph and Saint Luc Hospital. Prod Plan Control.

[120] Abo-Hamad W, Arisha A (2012). Multi-criteria framework for emergency department in Irish hospital. Proceedings of the winter simulation conference.

[121] Abo-Hamad W, Arisha A (2013). Simulation-based framework to improve patient experience in an emergency department. Eur J Oper Res.

[122] Wang Y, Lee LH, Chew EP, Lam SSW, Low SK, Ong MEH (2015). Multi-objective optimization for a hospital inpatient flow process via discrete event simulation. Proceedings of the winter simulation conference.

[123] Zhao Y, Peng Q, Strome T, Weldon E, Zhang M, Chochinov A (2015). Bottleneck detection for improvement of emergency department efficiency. Bus Process Manag J.

[124] Rashwan W, Habib H, Arisha A, Courtney G, Kennelly S (2016). An integrated approach of multi-objective optimization model for evaluating new supporting program in Irish hospitals. Proceedings of the winter simulation conference.

[125] Rashwan W, Arisha A (2015). Modeling behavior of nurses in clinical medical unit in university hospital: burnout implications. Proceedings of the winter simulation conference.

[126] Bair AE, Song WT, Chen Y, Morris BA (2009). The impact of inpatient boarding on emergency department crowding: a discrete-event simulation study. Proceedings of the spring computer simulation conference.

[127] Bountourelis T, Eckman D, Luangkesorn L, Schaefer A, Nabors SG, Clermont G (2012). Sensitivity analysis of an ICU simulation model. Proceedings of the winter simulation conference.

[128] Hamrock E, Paige K, Parks J, Scheulen J, Levin S (2013). Discrete event simulation for healthcare organizations: a tool for decision making. J Healthc Manag.

[129] Centeno MA, Albacete C, Terzano DO, Carrillo M, Ogazon T (2000). Project and process improvements in healthcare organizations: a simulation study of the radiology department at JMH. Proceedings of the winter simulation conference.

[130] Ramis FJ, Palma JL, Baesler FF (2001). The use of simulation for process improvement at an ambulatory surgery center. Proceedings of the winter simulation conference.

[131] Pasin F, Jobin MH, Cordeau JF (2002). An application of simulation to analyse resource sharing organisations among health-care organisations. Int J Oper Prod Manag.

[132] Wiinamaki A, Dronzek R (2003). Using simulation in the architectural concept phase of an emergency department design. Proceedings of the winter simulation conference.

[133] Samaha S, Armel WS, Starks DW (2003). The use of simulation to reduce the length of stay in an emergency department. Proceedings of the winter simulation conference.

[134] Blasak RE, Starks DW, Armel WS, Hayduk MC (2003). Healthcare process analysis: the use of simulation to evaluate hospital operations between the emergency department and a medical telemetry unit. Proceedings of the winter simulation conference.

[135] Baesler FF, Jahnsen HE, DaCosta M (2003). The use of simulation and design of experiments for estimating maximum capacity in an emergency room. Proceedings of the winter simulation conference.

[136] Centeno MA, Giachetti R, Linn R, Ismail AM (2003). A simulation-ilp based tool for scheduling ER staff. Proceedings of the winter simulation conference.

[137] Schenk JR, Huang D, Zheng N, Allen TT (2005). Multiple fidelity simulation optimization of hospital performance under high consequence event scenarios. Proceedings of the winter simulation conference.

[138] Spry CW, Lawley MA (2005). Evaluating hospital pharmacy staffing and work scheduling using simulation. Proceedings of the winter simulation conference.

[139] Hay AM, Valentin EC, Bijlsma RA (2006). Modeling emergency care in hospitals: a paradox-the patient should not drive the process. Proceedings of the winter simulation conference.

[140] Wijewickrama AKA, Takakuwa S (2006). Simulation analysis of an outpatient department of internal medicine in a university hospital. Proceedings of the winter simulation conference.

[141] Ballard SM, Kuhl ME (2006). The use of simulation to determine maximum capacity in the surgical suite operating room. Proceedings of the winter simulation conference.

[142] Taaffe K, Johnson M, Steinmann D (2006). Improving hospital evacuation planning using simulation. Proceedings of the winter simulation conference.

[143] VanBerkel PT, Blake JT (2007). A comprehensive simulation for wait time reduction and capacity planning applied in general surgery. Health Care Manag Sci.

[144] Rico F, Salari E, Centeno G (2007). Emergency departments nurse allocation to face a pandemic influenza outbreak. Proceedings of the winter simulation conference.

[145] Miller M, Ferrin D, Ashby M, Flynn T, Shahi N (2007). Merging six emergency departments into one: a simulation approach. Proceedings of the winter simulation conference.

[146] Patvivatsiri L, Montes EJ, Xi O (2007). Modeling bioterrorism preparedness with simulation in rural healthcare system. Proceedings of the winter simulation conference.

[147] Paul JA, Hariharan G (2007). Hospital capacity planning for efficient disaster mitigation during a bioterrorist attack. Proceedings of the winter simulation conference.

[148] Song WT, Bair AE, Chih M (2008). A simulation study on the impact of physician starting time in a physical examination service. Proceedings of the winter simulation conference.

[149] Protil RM, Stroparo JR, Bichinho GL (2008). Applying computer simulation to increase the surgical center occupation rate at a university hospital in Curitiba-Brazil. Proceedings of the winter simulation conference.

[150] Nielsen AL, Hilwig H, Kissoon N, Teelucksingh S (2008). Discrete event simulation as a tool in optimization of a professional complex adaptive system. Stud Health Technol Inform.

[151] Huschka TR, Denton BT, Narr BJ, Thompson AC (2008). Using simulation in the implementation of an outpatient procedure center. Proceedings of the winter simulation conference.

[152] Oddoye JP, Jones DF, Tamiz M, Schmidt P (2009). Combining simulation and goal programming for healthcare planning in a medical assessment unit. Eur J Oper Res.

[153] Raunak M, Osterweil L, Wise A, Clarke L, Henneman P (2009). Simulating patient flow through an emergency department using process-driven discrete event simulation. Proceedings of the international conference on software engineering.

[154] Efe K, Raghavan V, Choubey S (2009). Simulation modeling movable hospital assets managed with RFID sensors. Proceedings of the winter simulation conference.

[155] Ferrand Y, Magazine M, Rao U (2010). Comparing two operating-room-allocation policies for elective and emergency surgeries. Proceedings of the winter simulation conference.

[156] Zeltyn S, Marmor YN, Mandelbaum A, Carmeli B, Greenshpan O, Mesika Y (2011). Simulation-based models of emergency departments: operational, tactical, and strategic staffing. ACM Trans Model Comput Simul.

[157] Weng S-J, Tsai B-S, Wang L-M, Chang C-Y, Gotcher D (2011). Using simulation and data envelopment analysis in optimal healthcare efficiency allocations. Proceedings of the winter simulation conference.

[158] Geis GL, Pio B, Pendergrass TL, Moyer MR, Patterson MD (2011). Simulation to assess the safety of new healthcare teams and new facilities. Simul Healthc.

[159] Cabrera E, Luque E, Taboada M, Epelde F, Iglesias ML (2012). ABMS optimization for emergency departments. Proceedings of the winter simulation conference.

[160] Kuo Y-H, Leung JM, Graham CA (2012). Simulation with data scarcity: developing a simulation model of a hospital emergency department. Proceedings of the winter simulation conference.

[161] Mustafee N, Lyons T, Rees P, Davies L, Ramsey M, Williams MD (2012). Planning of bed capacities in specialized and integrated care units: incorporating bed blockers in a simulation of surgical throughput. Proceedings of the winter simulation conference.

[162] Rashwan W, Ragab M, Abo-Hamad W, Arisha A (2013). Evaluating policy interventions for delayed discharge: a system dynamics approach. Proceedings of the winter simulation conference.

[163] Shin SY, Balasubramanian H, Brun Y, Henneman PL, Osterweil LJ (2013). Resource scheduling through resource-aware simulation of emergency departments. Proceedings of the international workshop on software engineering in health care.

[164] Amyot D (2013). Real-time simulations to support operational decision making in healthcare. Proceedings of the summer computer simulation conference.

[165] Verma S, Gupta A (2013). Improving services in outdoor patient departments by focusing on process parameters: a simulation approach. Proceedings of the winter simulation conference.

[166] Yaylali E, Simmons J, Taheri J (2014). Systems engineering methods for enhancing the value stream in public health preparedness: the role of Markov models, simulation, and optimization. Public Health Rep.

[167] Pinto LR, Perpétuo IHO, de Campos FCC, Ribeiro YCNMB (2014). Analysis of hospital bed capacity via queuing theory and simulation. Proceedings of the winter simulation conference.

[168] Ozen A, Balasubramanian H, Samra P, Ehresman M, Li H, Fairman T (2014). The impact of hourly discharge rates and prioritization on timely access to inpatient beds. Proceedings of the winter simulation conference.

[169] Kalbasi A, Krishnamurthy D, Rolia J, Singhal S (2014). Simulation by example for complex systems. Proceedings of the winter simulation conference.

[170] Wurzer G, Lorenz WE (2014). Causality in hospital simulation based on utilization chains. Proceedings of the symposium on simulation for architecture and urban design.

[171] Aboueljinane L, Sahin E, Jemai Z, Marty J (2014). A simulation study to improve the performance of an emergency medical service: application to the French Val-de-Marne department. Simul Model Pract Theory.

[172] Ghanes K, Jouini O, Jemai Z, Wargon M, Hellmann R, Thomas V (2014). A comprehensive simulation modeling of an emergency department: a case study for simulation optimization of staffing levels. Proceedings of the winter simulation conference.

[173] Zhou Z, Wang Y, Li L (2014). Process mining based modeling and analysis of workflows in clinical care—a case study in a Chicago outpatient clinic. Proceedings of the IEEE international conference on networking, sensing and control.

[174] van Buuren M, Kommer GJ, van der Mei R, Bhulai S (2015). A simulation model for emergency medical services call centers. Proceedings of the winter simulation conference.

[175] Franck T, Augusto V, Xie X, Gonthier R, Achour E (2015). Performance evaluation of an integrated care for geriatric departments using discrete-event simulation. Proceedings of the winter simulation conference.

[176] Ghanes K, Wargon M, Jouini O, Jemai Z, Diakogiannis A, Hellmann R (2015). Simulation-based optimization of staffing levels in an emergency department. Simulation.

[177] Carmen R, Defraeye M, Van Nieuwenhuyse I (2015). A decision support system for capacity planning in emergency departments. Int J Simul Model.

[178] dos Santos M, Quintal RS, da PAC, Gomes CFS (2015). Simulation of operation of an integrated information for emergency pre-hospital care in Rio de Janeiro municipality. Procedia Comput Sci.

[179] Agor J, McKenzie K, Ozaltin O, Mayorga M, Parikh RS, Huddleston J (2016). Simulation of triaging patients into an internal medicine department to validate the use of an optimization based workload score. Proceedings of the winter simulation conference.

[180] Lee W, Shin K, Lee H-R, Shin H, Lee T (2016). A structured approach for constructing high fidelity ED simulation. Proceedings of the winter simulation conference.

[181] Pujowidianto NA, Lee LH, Pedrielli G, Chen C-H, Li H (2016). Constrained optimizaton for hospital bed allocation via discrete event simulation with nested partitions. Proceedings of the winter simulation conference.

[182] Augusto V, Xie X, Prodel M, Jouaneton B, Lamarsalle L (2016). Evaluation of discovered clinical pathways using process mining and joint agent-based discrete-event simulation. Proceedings of the winter simulation conference.

[183] Tiwari V, Sandberg WS (2016). Perioperative bed capacity planning guided by theory of constraints. Proceedings of the winter simulation conference.

[184] Thorwarth M, Rashwan W, Arisha A (2016). An analytical representation of flexible resource allocation in hospitals. Flex Serv Manuf J.

[185] Kuo Y-H, Rado O, Lupia B, Leung JMY, Graham CA (2016). Improving the efficiency of a hospital emergency department: a simulation study with indirectly imputed service-time distributions. Flex Serv Manuf J.

[186] Kadı D, Kuvvetli Y, Çolak S (2016). Performance analysis of a university hospital blood laboratory via discrete event simulation. Simulation.

[187] Cimellaro GP, Piqué M (2016). Resilience of a hospital emergency department under seismic event. Adv Struct Eng.

[188] Zhong X, Lee HK, Williams M, Kraft S, Sleeth J, Welnick R, Matta A, Sahin E, Li J, Guinet A, Vandaele NJ (2016). Staffing ratio analysis in primary care redesign: a simulation approach. Health care systems engineering for scientists and practitioners.

[189] Lambton J, Roeder T, Saltzman R, Param L, Fernandes R (2017). Using simulation to model improvements in pediatric bed placement in an acute care hospital. Jona J Nurs Adm.

[190] Bakker M, Tsui K-L (2017). Dynamic resource allocation for efficient patient scheduling: a data-driven approach. J Syst Sci Syst Eng.

[191] Weng S-J, Xu Y-Y, Gotcher D, Wang L-M (2017). A pilot study of available bed forecasting system (ABFS) in the emergency healthcare network. Proceedings of the summer computer simulation conference.

[192] Tànfani E, Testi A (2010). Improving surgery department performance via simulation and optimization. Proceedings of the IEEE workshop on health care management.

[193] Friemann F, Schönsleben P (2016). Reducing global supply chain risk exposure of pharmaceutical companies by further incorporating warehouse capacity planning into the strategic supply chain planning process. J Pharm Innov.

[194] Ramis FJ, Baesler F, Berho E, Neriz L, Sepulveda JA (2008). A simulator to improve waiting times at a medical imaging center. Proceedings of the winter simulation conference.

[195] Zeinali F, Mahootchi M, Sepehri MM (2015). Resource planning in the emergency departments: a simulation-based metamodeling approach. Simul Model Pract Theory.

[196] Wang J, Zhong X, Li J, Howard PK (2014). Modeling and analysis of care delivery services within patient rooms: a system-theoretic approach. IEEE Trans Autom Sci Eng.

[197] Luangkesorn KL, Bountourelis T, Schaefer A, Nabors S, Clermont G (2012). The case against utilization: deceptive performance measures in inpatient care capacity models. Proceedings of the winter simulation conference.

[198] Holm LB, Dahl FA (2010). Simulating the influence of a 45% increase in patient volume on the emergency department of Akershus University Hospital. Proceedings of the winter simulation conference.

[199] Miller MJ, Ferrin DM, Szymanski JM (2003). Simulating six sigma improvement ideas for a hospital emergency department. Proceedings of the winter simulation conference.

[200] Mackay M, Qin S, Clissold A, Hakendorf P, Ben-Tovim D, McDonnell G (2013). Patient flow simulation modelling-an approach conducive to multi-disciplinary collaboration towards hospital capacity management. Proceedings of the international congress on modelling and simulation.

[201] Mes M, Bruens M (2012). A generalized simulation model of an integrated emergency post. Proceedings of the winter simulation conference.

[202] Khurma N, Bacioiu GM, Pasek ZJ (2008). Simulation-based verification of lean improvement for emergency room process. Proceedings of the winter simulation conference.

[203] Centeno MA, Lee MA, Lopez E, Fernandez HR, Carrillo M, Ogazon T (2001). A simulation study of the labor and delivery rooms at JMH. Proceeding of the winter simulation conference.

[204] Ramakrishnan S, Nagarkar K, DeGennaro M, Srihari K, Courtney AK, Emick F (2004). A study of the CT scan area of a healthcare provider. Proceedings of the winter simulation conference.

[205] Ruohonen T, Neittaanmäki P, Teittinen J (2006). Simulation model for improving the operation of the emergency department of special health care. Proceedings of the winter simulation conference.

[206] Bountourelis T, Luangkesorn L, Schaefer A, Maillart L, Nabors SG, Clermont G (2011). Development and validation of a large scale ICU simulation model with blocking. Proceedings of the winter simulation conference.

[207] Al-Araidah O, Boran A, Wahsheh A (2012). Reducing delay in healthcare delivery at outpatients clinics using discrete event simulation. Int J Simul Model.

[208] Pasupathy R (2013). Performance evaluation in a laboratory medicine unit. Proceedings of the winter simulation conference.

[209] Taylor SJE, Abbott P, Young T, Grocott-Mason R (2014). Student modeling & simulation projects in healthcare: experiences with Hillingdon Hospital. Proceedings of the winter simulation conference.

[210] Oh C, Novotny AM, Carter PL, Ready RK, Campbell DD, Leckie MC (2016). Use of a simulation-based decision support tool to improve emergency department throughput. Oper Res Health Care.

[211] Eskandari H, Riyahifard M, Khosravi S, Geiger CD (2011). Improving the emergency department performance using simulation and MCDM methods. Proceedings of the winter simulation conference.

[212] Mielczarek B, Uziałko J (2012). Using simulation to forecast the demand for hospital emergency services at the regional level. Proceedings of the winter simulation conference.

[213] Perimal-Lewis L (2014). Health intelligence: discovering the process model using process mining by constructing start-to-end patient journeys. Proceedings of the Australasian workshop on health informatics and knowledge management.

[214] Hosseini N, Taaffe K (2014). Evaluation of optimal scheduling policy for accommodating elective and non-elective surgery via simulation. Proceedings of the winter simulation conference.

[215] Esengul Tayfur TK (2007). Allocation of resources for hospital evacuations via simulation. Proceedings of the winter simulation conference.

[216] Ashby M, Miller M, Ferrin D, Flynn T (2007). Simulating the patient move: transitioning to a replacement hospital. Proceedings of the winter simulation conference.

[217] Pérez ES, Yepez LA, de la Mota IF (2010). Simulation and optimization of the pre-hospital care system of the National University of Mexico using travelling salesman problem algorithms. Proceedings of the summer computer simulation conference.

[218] Güttinger D, Godehardt E, Zinnen A (2011). Optimizing emergency supply for mass events. Proceedings of the 4th international ICST (Institute for Computer Sciences, social-informatics and telecommunications engineering) conference on simulation tools and techniques.

[219] Noreña D, Yamín L, Akhavan-Tabatabaei R, Ospina W (2011). Using discrete event simulation to evaluate the logistics of medical attention during the relief operations in an earthquake in Bogota. Proceedings of the winter simulation conference.

[220] Ullrich C, Van Utterbeeck F, Dejardin E, Debacker M, Dhondt E (2013). Pre-hospital simulation model for medical disaster management. Proceedings of the winter simulation conference.

[221] Noei S, Santana H, Sargolzaei A, Noei M (2014). Reducing traffic congestion using geo-fence technology: application for emergency car. Proceedings of the international workshop on emerging multimedia applications and services for smart cities.

[222] Moon I-C, Bae JW, Lee J, Kim D, Lee H, Lee T (2015). EMSSIM: emergency medical service simulator with geographic and medical details. Proceedings of the winter simulation conference.

[223] Gibson IW (2007). An approach to hospital planning and design using discrete event simulation. Proceedings of the winter simulation conference.

[224] Miller MJ, Ferrin DM, Shahi N, LaVecchia R (2008). Allocating outpatient clinic services using simulation and linear programming. Proceedings of the winter simulation conference.

[225] Ashby M, Ferrin D, Miller M, Shahi N (2008). Discrete event simulation: optimizing patient flow and redesign in a replacement facility. Proceedings of the winter simulation conference.

[226] Boucherie RJ, Hans EW, Hartmann T (2012). Health care logistics and space: accounting for the physical build environment. Proceedings of the winter simulation conference.

[227] Wurzer G (2013). In-process agent simulation for early stages of hospital planning. Math Comput Model Dyn Syst.

[228] Wurzer G, Lorenz WE, Rössler M, Hafner I, Popper N, Glock BMODYPLAN (2015). Early-stage hospital simulation with emphasis on cross-clinical treatment chains. Proceedings of the symposium on simulation for architecture and urban design.

[229] Schaumann D, Pilosof NP, Date K, Kalay YE (2016). A study of human behavior simulation in architectural design for healthcare facilities. Ann Dell Ist Super Sanita.

[230] Cimellaro GP, Malavisi M, Mahin S (2017). Using discrete event simulation models to evaluate resilience of an emergency department. J Earthq Eng.

[231] Pulat PS, Kasap S, Splinter GL (2001). Simulation study of an ideal primary care delivery system. Simulation.

[232] Moeke D, van de Geer R, Koole G, Bekker R (2016). On the performance of small-scale living facilities in nursing homes: a simulation approach. Oper Res Health Care.

[233] Maroufkhani A, Lanzarone E, Castelnovo C, Di Mascolo M, Matta A, Sahin E, Li J, Guinet A, Vandaele NJ (2016). A discrete event simulation model for the admission of patients to a home care rehabilitation service. Health care systems engineering for scientists and practitioners.

[234] Bhattacharjee P, Ray PK (2014). Patient flow modelling and performance analysis of healthcare delivery processes in hospitals: a review and reflections. Comput Ind Eng.

[235] Hu S, Heim JA (2014). Developing domain-specific simulation objects for modeling clinical laboratory operations. Proceedings of the winter simulation conference.

[236] Workman RW (2000). Simulation of the drug development process: a case study from the pharmaceutical industry. Proceedings of the winter simulation conference.

[237] Blau GE, Pekny JF, Varma VA, Bunch PR (2004). Managing a portfolio of interdependent new product candidates in the pharmaceutical industry. J Prod Innov Manag.

[238] Chen Y, Mockus L, Orcun S, Reklaitis GV (2012). Simulation-optimization approach to clinical trial supply chain management with demand scenario forecast. Comput Chem Eng.

[239] Perez-Escobedo JL, Azzaro-Pantel C, Pibouleau L (2012). Multiobjective strategies for New Product Development in the pharmaceutical industry. Comput Chem Eng.

[240] Chen Y, Pekny JF, Reklaitis GV (2013). Integrated planning and optimization of clinical trial supply chain system with risk pooling. Ind Eng Chem Res.

[241] El Afia A, Mezouar H (2017). A global mapping of the Moroccan supply chain of hospital drugs, and a simulation of the dispensation process. Proceedings of the international conference on big data, cloud and applications.

[242] Huyghe J, Nouwen M, Vanattenhoven J (2016). Involving end-users in game based ideation: a case study in hospital logistics. Proceedings of the Nordic conference on human-computer interaction.

[243] Best AM, Dixon CA, Kelton WD, Lindsell CJ, Ward MJ (2014). Using discrete event computer simulation to improve patient flow in a Ghanaian acute care hospital. Am J Emerg Med.

[244] Caputo AC, Pelagagge PM (2006). Management criteria of automated order picking systems in high-rotation high-volume distribution centers. Ind Manag Data Syst.

[245] Katsaliaki K, Brailsford SC (2007). Using simulation to improve the blood supply chain. J Oper Res Soc.

[246] Vila-Parrish AR, Ivy JS, King RE (2008). A simulation-based approach for inventory modeling of perishable pharmaceuticals. Proceedings of the winter simulation conference.

[247] Jung JY, Blau G, Pekny JF, Reklaitis GV, Eversdyk D (2008). Integrated safety stock management for multi-stage supply chains under production capacity constraints. Comput Chem Eng.

[248] Mustafee N, Taylor SJE, Katsaliaki K, Brailsford S (2009). Facilitating the analysis of a UK national blood service supply chain using distributed simulation. Simulation.

[249] Rossetti MD, Liu Y (2009). Simulating SKU proliferation in a health care supply chain. Proceedings of the winter simulation conference.

[250] Ren C, Wang W, He M, Shao B, Wang Q, Dong J. The use of simulation for Global Supply Network rationalization. In: Proceedings of IEEE international conference on service operations and logistics, and informatics. 2010. p. 276–81.

[251] Babaï MZ, Syntetos AA, Dallery Y, Nikolopoulos K (2009). Dynamic re-order point inventory control with lead-time uncertainty: analysis and empirical investigation. Int J Prod Res.

[252] Baesler F, Bastías A, Nemeth M, Martínez C (2012). Blood centre inventory analysis using discrete simulation. Proceedings of the winter simulation conference.

[253] Onggo BS (2014). Elements of a hybrid simulation model: a case study of the blood supply chain in low-and middle-income countries. Proceedings of the winter simulation conference.

[254] Gebicki M, Mooney E, Chen S-J, Mazur LM (2014). Evaluation of hospital medication inventory policies. Health Care Manag Sci.

[255] Duan Q, Liao TW (2014). Optimization of blood supply chain with shortened shelf lives and ABO compatibility. Int J Prod Econ.

[256] Baesler F, Nemeth M, Martínez C, Bastías A (2014). Analysis of inventory strategies for blood components in a regional blood center using process simulation. Transfusion.

[257] Wang K-M, Ma Z-J (2015). Age-based policy for blood transshipment during blood shortage. Transp Res Part E Logist Transp Rev.

[258] Leung N-HZ, Chen A, Yadav P, Gallien J (2016). The impact of inventory management on stock-outs of essential drugs in Sub-Saharan Africa: secondary analysis of a field experiment in Zambia. PLoS One.

[259] Yurtkuran A, Emel E (2008). Simulation based decision-making for hospital pharmacy management. Proceedings of the winter simulation conference.

[260] Lee YM (2008). Analyzing dispensing plan for emergency medical supplies in the event of bioterrorism. Proceedings of the winter simulation conference.

[261] Lee YM, Ghosh S, Ettl M (2009). Simulating distribution of emergency relief supplies for disaster response operations. Proceedings of the winter simulation conference.

[262] Ozdamar L (2011). Planning helicopter logistics in disaster relief. Spectrum.

[263] Kulkarni NS, Niranjan S (2013). Multi-echelon network optimization of pharmaceutical cold chains: a simulation study. Proceedings of the winter simulations conference.

[264] Postacchini L, Ciarapica FE, Bevilacqua M, Mazzuto G, Paciarotti C (2016). A way for reducing drug supply chain cost for a hospital district: a case study. J Ind Eng Manag.

[265] Abdelkafi C, Beck BHL, David B, Druck C, Horoho M (2009). Balancing risk and costs to optimize the clinical supply chain-a step beyond simulation. J Pharm Innov.

[266] Alfonso E, Xie X, Augusto V, Garraud O (2013). Modelling and simulation of blood collection systems: improvement of human resources allocation for better cost-effectiveness and reduction of candidate donor abandonment. Vox Sang.

[267] Cho S-H, Jang H, Lee T, Turner J (2014). Simultaneous location of trauma centers and helicopters for emergency medical service planning. Oper Res.

[268] Liao H-C, Chang H-H (2011). The optimal approach for parameter settings based on adjustable contracting capacity for the hospital supply chain logistics system. Expert Syst Appl.

[269] Akcay A, Martagan T (2016). Stochastic simulation under input uncertainty for contract-manufacturer selection in pharmaceutical industry. Proceedings of the winter simulation conference.

[270] Jacobs EA, Bickel WK (1999). Modeling drug consumption in the clinic using simulation procedures: demand for heroin and cigarettes in opioid-dependent outpatients. Exp Clin Psychopharmacol.

[271] Royston G, Dost A, Townshend J, Turner H (1999). Using system dynamics to help develop and implement policies and programmes in health care in England. Syst Dyn Rev.

[272] Mustafee N, Taylor SJE, Katsaliaki K, Brailsford S (2006). Distributed simulation with COTS simulation packages: a case study in health care supply chain simulation. Proceedings of the winter simulation conference.

[273] Muller J, Popke C, Urbat M, Zeier A, Plattner H (2009). A simulation of the pharmaceutical supply chain to provide realistic test data. Proceedings of the international conference on advances in system simulation.

[274] Devi SP, Rao KS, Krishnaswamy S, Wang S (2010). System dynamics model for simulation of the dynamics of corneal transplants. OPSEARCH.

[275] Ng Adam TS, Sy C, Li J (2011). A system dynamics model of Singapore healthcare affordability. Proceedings of the winter simulation conference.

[276] Djanatliev A, German R, Kolominsky-Rabas P, Hofmann BM (2012). Hybrid simulation with loosely coupled system dynamics and agent-based models for prospective health technology assessments. Proceedings of the winter simulation conference.

[277] zen MB (2013). Estimating future demand for hospital emergency services at the regional level. Proceedings of the winter simulation conference.

[278] Elleuch H, Hachicha W, Chabchoub H (2014). A combined approach for supply chain risk management: description and application to a real hospital pharmaceutical case study. J Risk Res.

[279] Padilla JJ, Diallo SY, Kavak H, Sahin O, Sokolowski JA, Gore RJ (2015). Semi-automated initialization of simulations: an application to healthcare. J Def Model Simul.

[280] Taaffe K, Zinouri N, Kamath AG (2016). Integrating simulation modeling and mobile technology to improve day-of-surgery patient care. Proceedings of the winter simulation conference.

[281] Pitt M, Monks T, Crowe S, Vasilakis C (2016). Systems modelling and simulation in health service design, delivery and decision making. BMJ Qual Saf.

[282] Jahangirian M, Borsci S, Shah SGS, Taylor SJE (2015). Causal factors of low stakeholder engagement: a survey of expert opinions in the context of healthcare simulation projects. Simulation.

[283] Dangerfield BC (1999). System dynamics applications to European health care issues. J Oper Res Soc.

[284] Harrell CR, Price RN (2000). Healthcare simulation modeling and optimization using MedModel. Proceedings of the winter simulation conference.

[285] Joustra P, van der Sluis E, van Dijk NM (2010). To pool or not to pool in hospitals: a theoretical and practical comparison for a radiotherapy outpatient department. Ann Oper Res.

[286] Morrison BP, Bird BC (2003). Healthcare process analysis: a methodology for modeling front office and patient care processes in ambulatory health care. Proceedings of the winter simulation conference.

[287] Baldwin LP, Eldabi T, Paul RJ (2004). Simulation in healthcare management: a soft approach (MAPIU). Simul Model Pract Theory.

[288] White KP (2005). A survey of data resources for simulating patient flows in healthcare delivery systems. Proceedings of the winter simulation conference.

[289] Brailsford S (2005). Overcoming the barriers to implementation of operations research simulation models in healthcare. Clin Investig Med Med Clin Exp.

[290] Eldabi T, Paul RJ, Young T (2007). Simulation modelling in healthcare: reviewing legacies and investigating futures. J Oper Res Soc.

[291] Chahal K, Eldabi T (2008). Applicability of hybrid simulation to different modes of governance in UK healthcare. Proceedings of the winter simulation conference.

[292] Gupta D, Denton B (2008). Appointment scheduling in health care: challenges and opportunities. IIE Trans.

[293] Brailsford SC, Harper PR, Patel B, Pitt M (2009). An analysis of the academic literature on simulation and modelling in health care. J Simul.

[294] Katsaliaki K, Mustafee N, Taylor SJE, Brailsford S (2009). Comparing conventional and distributed approaches to simulation in a complex supply-chain health system. J Oper Res Soc.

[295] Forsythe L (2009). Action research, simulation, team communication, and bringing the tacit into voice society for simulation in healthcare. Simul Healthc.

[296] Seropian M, Lavey R (2010). Design considerations for healthcare simulation facilities. Simul Healthc.

[297] Katsaliaki K, Mustafee N (2011). Applications of simulation within the healthcare context. J Oper Res Soc.

[298] LeBlanc VR, Manser T, Weinger MB, Musson D, Kutzin J, Howard SK (2011). The study of factors affecting human and systems performance in healthcare using simulation. Simul Healthc.

[299] Beliën J, Forcé H (2012). Supply chain management of blood products: a literature review. Eur J Oper Res.

[300] Jahangirian M, Naseer A, Stergioulas L, Young T, Eldabi T, Brailsford S (2012). Simulation in health-care: lessons from other sectors. Oper Res.

[301] Hong TS, Shang PP, Arumugam M, Yusuff RM (2013). Use of simulation to solve outpatient clinic problems: a review of the literature. South Afr J Ind Eng.

[302] Holm LB, Dahl FA (2009). Simulating the effect of physician triage in the emergency department of Akershus University Hospital. Proceedings of the winter simulation conference.

[303] Günal MM, Pidd M (2008). DGHPSim: supporting smart thinking to improve hospital performance. Proceedings of the winter simulation conference.

[304] Yeon N, Lee T, Jang H (2010). Outpatients appointment scheduling with multi-doctor sharing resources. Proceedings of the winter simulation conference.

[305] Sugiyama T, Goryoda S, Inoue K, Sugiyama-Ihana N, Nishi N (2017). Construction of a simulation model and evaluation of the effect of potential interventions on the incidence of diabetes and initiation of dialysis due to diabetic nephropathy in Japan. BMC Health Serv Res.

