# Correction to: A systematic literature review of simulation models for non-technical skill training in healthcare logistics

**DOI:** 10.1186/s41077-019-0094-9

**Published:** 2019-04-24

**Authors:** Cevin Zhang, Thomas Grandits, Karin Pukk Härenstam, Jannicke Baalsrud Hauge, Sebastiaan Meijer

**Affiliations:** 1 School of Engineering Sciences in Chemistry, Biotechnology and Health, Royal Institute of Technology, 2010, Röntgenvägen 1, 14152 Huddinge, Sweden; 2 School of Engineering Sciences in Chemistry, Biotechnology and Health, Royal Institute of Technology, Hälsovägen 11, 14152 Huddinge, Sweden; 3 Pediatric Emergency Department, Karolinska University Hospital, Tomtebodavägen 18a, 17177 Stockholm, Sweden; 4 Department of Learning, Informatics, Management and Ethics, Karolinska Institute, Tomtebodavägen 18a, 17177 Stockholm, Sweden; 5 School of Industrial Engineering and Management, Royal Institute of Technology, Mariekällgatan 3, 15144 Södertälje, Sweden

**Correction to: Adv Simul**

**https://doi.org/10.1186/s41077-018-0072-7**

The original article [1] contains a previous iteration of author, Chen Zhang’s name. This should instead be displayed as Cevin Zhang as shown in this Correction article.
